# Validity and reliability of the Chinese version of the Young Positive Schema Questionnaire

**DOI:** 10.3389/fpsyg.2022.1048954

**Published:** 2022-12-02

**Authors:** Danni Chi, Xiangju Du, Hong Ma, Yubo Wang, Yuanyuan Zhang, Haiyun Zhong

**Affiliations:** Department of Psychosomatic Medicine, Ningbo Kangning Hospital, Ningbo, China

**Keywords:** early adaptive schema, positive schema, resilience, life satisfaction, depression, anxiety

## Abstract

**Introduction:**

The Young Positive Schema Questionnaire (YPSQ) examines early adaptive or positive schemas as a counterpart to early maladaptive ones. This study investigated the validity and reliability of the Chinese version of the YPSQ (CYPSQ).

**Methods:**

A convenient community sample of 634 individuals, most of whom were college students, were recruited through a mobile survey. R Careless was used to screen for careless responses. A final sample of 336 was obtained. Confirmatory factor analyses (CFA) of the CYPSQ were conducted using SPSS AMOS 25. Other statistical analyses were conducted using SPSS 25.

**Results:**

An 11-factor CYPSQ was identified with an acceptable factor structure (χ^2^/df = 2.13, SRMR = 0.04, RMSEA = 0.06, GFI = 0.80, TLI = 0.90, and CFI = 0.91). Convergent and discriminant validity were confirmed in most aspects. Concurrent validity was evident with resilience, life satisfaction, depression, and anxiety. Internal reliability was satisfied as the Cronbach’s alphas of the 11 factors of the CYPSQ ranged between 0.70 and 0.88.

**Conclusion:**

The findings supported the reliability and validity of the CYPSQ in Mainland China.

## Introduction

Schema therapy is an integrative method that combines attachment theories and cognitive behavior therapy. It was proposed and developed by Young et al. (2003). A negative or early maladaptive schema (EMS) or negative schema refers to a broad and pervasive pattern of cognitions, memories, emotions, and physical sensations vis-à-vis oneself and one’s relationships with others, which is dysfunctional to a significant degree (Young et al., 2003). Negative schemas are the outcomes of unmet core emotional needs in childhood and/or adolescence (i.e., acceptance, autonomy, reasonable limits, realistic expectations, and connection) and are elaborated upon throughout one’s lifetime (Young et al., 2003). Empirical studies have found that negative schemas are the transdiagnostic mechanisms of personality and affective disorders (Wang et al., 2010; Bamelis et al., 2014; Peeters et al., 2021). However, individuals with maladaptive schemas in some areas can also have adaptive or positive schemas in other areas.

Traditional clinical psychology is criticized as overly focused on the psychopathology of mental issues, and increasing interest has been invested in positive clinical psychology in recent decades (Seligman et al., 2006; Wood and Tarrier, 2010; Rashid, 2015). Positive or early adaptive schemas are expected when a person’s core emotional needs are sufficiently met, which are counterparts of negative schemas (Lockwood and Perris, 2012). Like EMS, positive schemas comprise cognitions, memories, bodily sensations, and neurobiological reactions of individuals with both themselves, and others but are positive and functional (Lockwood and Perris, 2012). Positive schemas explained extra variance in psychopathological outcomes, life satisfaction, resilience, and self-efficacy after controlling for EMSs (Louis et al., 2018; Paetsch et al., 2020). They are unique constructs with the potential to improve one’s wellbeing and mental health. Therefore, it is important to evaluate positive schemas in clinical and general populations.

The Young Positive Schema Questionnaire (YPSQ, Louis et al., 2018) was developed within the schema therapy framework to assess 14 positive schemas in adults. As the concept and measurement of positive schemas were proposed a few years ago (Lockwood and Perris, 2012; Louis et al., 2018), the validity and reliability of the YPSQ are yet to be verified in Mainland China. Thus, the present study investigated the psychometric properties of the Chinese version of YPSQ (CYPSQ) in Mainland China.

## Materials and methods

### Research design

**FIGURE 1:**
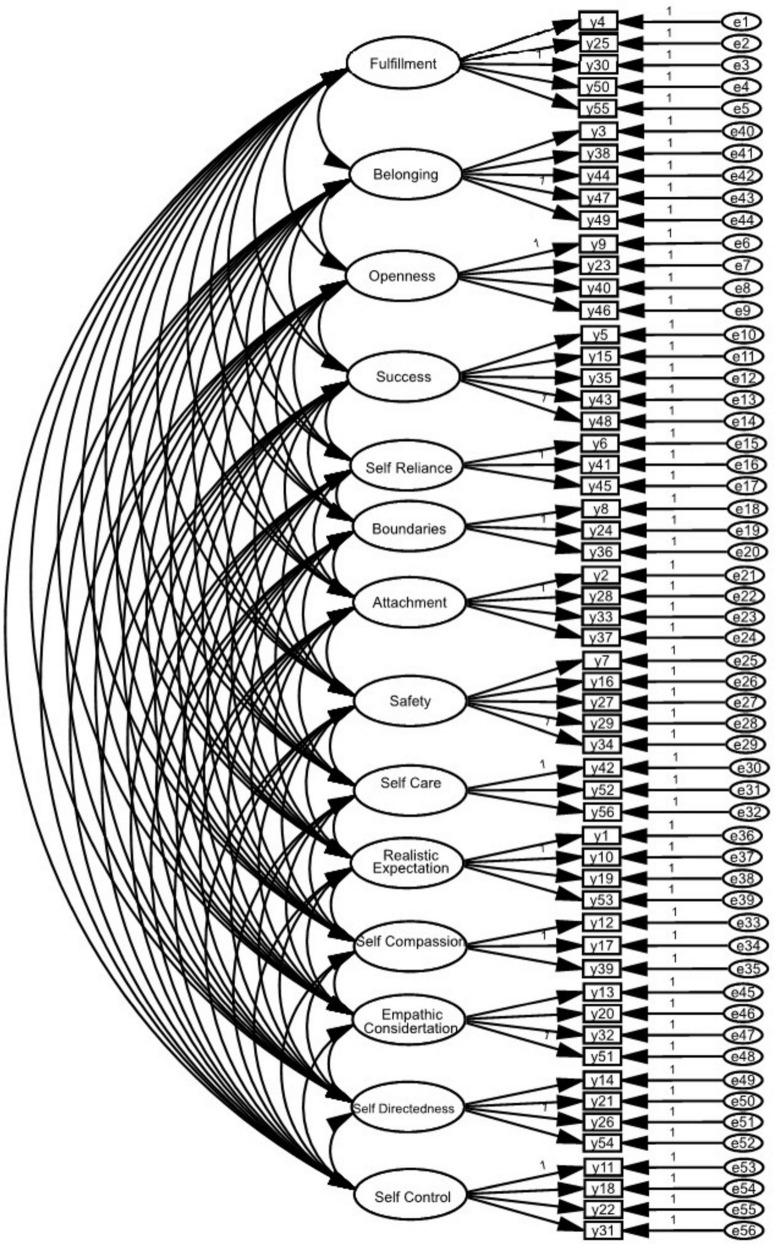
Factor construct of the 14-factor Young Positive Schema Questionnaire (YPSQ) (Louis et al., 2018).

This study used a cross-sectional design to evaluate the validity and reliability of CYPSQ. The original 56-item CYPSQ included 14 factors (Figure 1), according to Louis et al. (2018). In this study, the minimum desired sample size was 300 as it satisfied a ratio of five samples per item and was suggested as good for factor analysis (Comrey and Lee, 1992).

Permission to use and adjust the CYPSQ was obtained from the original author (Louis et al., 2018), who offered both English and Chinese versions of the YPSQ. A group of researchers, including a psychologist with a Ph.D in psychology in an English-speaking country and two psychiatrists with advanced levels of English proficiency, evaluated the appropriateness of CYPSQ. Minor adjustments were made to ensure that the scale was understandable in Mainland China. The original author was informed of the changes. After that, 10 nurses went through the CYPSQ and reported that there was no confusion.

### Ethical considerations

This study was completely anonymous and voluntary and was approved by the Institutional Ethics Committee of Ningbo Kangning Hospital (NBKNYY-2021-LC-50). Written consent was obtained from participants in accordance with the ethical standards of national laws. The study was conducted in accordance with the Declaration of Helsinki (Bošnjak, 2001; Tyebkhan, 2003; World Medical Association, 2013) and the Standards of Ethics in Sport and Exercise Science Research (Harriss et al., 2019).

### Participants

Recruitment took place from 16 February to 19 April, 2022. Recruitment information was delivered through an online survey platform that was accessed through a mobile phone. Snowball sampling was applied in recruiting the community participants. The inclusion criteria were: aged between 18 and 75 years, fluent in Chinese, and capable of understanding and answering the survey. The convenience sample comprised 634 individuals, most of whom were college students. After evaluating eligibility and screening the data for careless responses and outliers, a final sample of 336 individuals was obtained for the validity and reliability tests, which is adequate for a confirmatory factor analysis (CFA).

### Instruments

#### Demographics

Participants were asked to provide information on their age, gender, and educational background.

#### Young Positive Schema Questionnaire

The Young Positive Schema Questionnaire (YPSQ) is a self-report inventory comprising 14 factors and 56 items (Louis et al., 2018). Participants were asked to rate the degree to which the statements applied to them over the preceding month. Each item is rated on a 6-point Likert-type scale, ranging from 1 (completely untrue of me) to 6 (describes me perfectly). Higher scores indicate more positive schemas. The Cronbach’s α of each factor in the 14-factor original and 10-factor German YPSQ were above 0.70 (Louis et al., 2018; Paetsch et al., 2020). This was also the case in the 11-factor CYPSQ as seen in this study. The Cronbach’s α of the overall score of the 11-factor CYPSQ was 0.98 in this study.

#### Life satisfaction scale

The life satisfaction subscale from the Comprehensive Inventory of Thriving (CIT; Su et al., 2014) was used to measure life satisfaction over the preceding month. The original CIT was valid and reliable (Duan et al., 2020), and subscales could be used selectively (Su et al., 2014). This self-report life satisfaction subscale comprises three items, each of which is rated on a 5-point Likert-type scale, ranging from 1 (strongly disagree) to 5 (strongly agree). Higher scores indicate greater life satisfaction. The Cronbach’s α was 0.88 in this study.

#### Resilience scale

The Chinese version of the 10-item Connor–Davidson Resilience Scale (CD-RISK-10; Connor and Davidson, 2003) was used to measure the resilience level of participants over the preceding month. The CD-RISK-10 is a 5-point Likert-type scale, ranging from 0 (not true at all) to 4 (true nearly all the time). Higher scores indicate greater resilience. The CD-RISK-10 is a self-report scale and has been used among Chinese college students and shown good validity and reliability (Yu and Zhang, 2007; Cheng et al., 2020). The Cronbach’s α was 0.93 in this study.

#### Anxiety scale

The 7-item Generalized Anxiety Disorder (GAD-7; Spitzer et al., 2006) scale was used to examine the anxiety level of participants in this study. Participants were asked to rate their degree of anxiety over the preceding month. Each item is rated on a 4-point Likert-type scale, ranging from 0 (not at all) to 3 (nearly every day). Higher scores indicate higher levels of anxiety. The GAD-7 has good validity and reliability for screening anxiety in clinical practice and research. The Cronbach’s α of the GAD-7 was 0.92 in this study.

#### Depression scale

The 9-item Patient Health Questionnaire (PHQ-9; Kroenke et al., 2001) is a 9-item self-administered questionnaire and was used to measure depressive mood. Participants were asked to rate the degree of their depression over the preceding month. Each item was rated on a 4-point Likert-type scale, ranging from 0 (not at all) to 3 (nearly every day). Higher scores indicate higher levels of depression. The Cronbach’s α of the PHQ-9 was 0.91 in this study.

### Statistical analyses

The web-based recruitment method usually results in a relatively higher number of careless responses (Johnson, 2005). Several strategies were adopted to screen the outliers and careless responses as suggested (Johnson, 2005; Yentes and Wilhelm, 2018). Careless R package was used to test the maximum longstring and the Mahalanobis D outlier indices with a default significant level of *p* < 0.01 (Yentes and Wilhelm, 2018). There is no golden standard for the cut-off number of longstring index or response time. Some studies recommend a maximum of 14 longstrings (Costa and McCrae, 2008) and no less than 2 s per English item as appropriate (Huang et al., 2012), but there are no relevant references for Chinese scales. In this study, similar criteria were applied. Cases were removed if any of the following were present: longstrings larger than 16, mean response time for each item less than 2 s, flagged Mahalanobis D, and absolute *Z* scores larger than 3.29 of the 56-item YPSQ.

The demographics were analyzed using descriptive statistics. The scale scores were computed as the means of items. *T*-tests were used to examine the mean differences in the YPSQ based on gender. The missing completely at random test suggested that the missing values were completely at random (chi-square = 3,435.01, *p* = 1.00) and missing values were excluded using pairwise deletion. Bivariate correlations were examined with Pearson correlation. The significance level of this study was set at *p* < 0.05. The CFA was examined using SPSS AMOS 25. Goodness-of-fit indices (GFIs) used in this study included the standardized root-mean residual (SRMR), the root-mean square error of approximation (RMSEA), the GFI, the Comparative Fit Index (CFI), and the Tucker–Lewis Index (TLI). The RMSEA, CFI, and TLI were calculated with the original 336 data set. The GFI and SRMR were calculated with the imputed data sets, as the AMOS 25 did not provide GFI and SRMR in data sets with missing data. The GFI and SRMR in these imputed data sets were the same. Recommended indices, such as χ^2^/df between 2 and 3, SRMR ≤ 0.08, RMSEA ≤ 0.08, GFI ≥ 0.90, CFI ≥ 0.90, and TLI ≥ 0.90, were used (Hu and Bentler, 1999). Convergent validity was confirmed by examining the standardized factor loadings, construct or composite reliability, and average variance extracted (AVE) value. Composite reliability and AVE values greater than 0.70 and 0.50, respectively, were considered valid (Fornell and Larcker, 1981; Hair et al., 2014). Discriminant validity was verified using the correlation coefficients between factors and the root square of the AVE value of each factor, and concurrent validity was examined using Pearson’s correlation between the CYPSQ factors and measures of resilience, life satisfaction, depression, and anxiety. Reliability was confirmed using the criterion of Cronbach’s α > 0.70 (Nunnally and Bernstein, 1994).

## Results

This study examined the construct and concurrent validity, and internal reliability of CYPSQ in a Chinese community sample.

### Demographics

Table 1 presents the demographics of the participants. The mean age of the participants was 27.11 years (SD = 11.75), as the sample was mainly from a college community. Most participants (91.0%) received college-level education or above and were female (*n* = 247, 73.5%).

**TABLE 1:** Demographics of participants.

Demographics	Frequency	Percentage (%)
**Age**		
Mean (SD)	27.11 (11.75)	
18–20	202	60.1
21–30	18	5.4
31–40	59	17.6
41–50	39	11.6
51–60	12	3.6
61–70	3	0.9
Above 70	0	0
Missing	3	0.9
**Gender**		
Men	86	25.6
Women	247	73.5
Trans gender	3	0.9
**Education**		
Middle school and below	3	0.9
High school	27	8.0
College or University	277	82.4
Postgraduate and above	29	8.6

*N* = 336.

In the present study, women reported lower levels of success (*t* = 2.60, *p* = 0.01), competence (*t* = 3.10, *p* < 0.01), safety (*t* = 2.94, *p* < 0.01), self-compassion (*t* = 2.41, *p* = 0.02), and self-control (*t* = 2.95, *p* < 0.01) than men, and no significant differences between genders were found for other factors (Table 2). Age was positively correlated with all the 11 CYPSQ factors (Table 3).

**TABLE 2:** Gender differences on the 11-factor Chinese version of the Young Positive Schema Questionnaire (CYPSQ).

CYPSQ factor	Gender	*N*	Mean	SD	*t*	*p*	95% CI	
							
							Lower	Upper
Fulfillment	Male	86	4.70	0.78	0.09	0.93	−0.19	0.21
	Female	247	4.69	0.84				
Belonging	Male	86	4.63	0.68	1.85	0.07	−0.01	0.36
	Female	247	4.45	0.77				
Openness	Male	86	4.54	0.82	1.64	0.10	−0.04	0.39
	Female	247	4.36	0.89				
Success	Male	86	4.54	0.82	2.60	0.01	0.06	0.45
	Female	247	4.28	0.78				
Competence	Male	86	4.94	0.84	3.10	<0.01	0.12	0.55
	Female	247	4.61	0.87				
Attachment	Male	86	4.55	0.89	1.85	0.07	−0.01	0.45
	Female	247	4.33	0.95				
Safety	Male	86	4.41	0.87	2.94	<0.01	0.10	0.52
	Female	247	4.10	0.83				
Care	Male	86	4.89	0.74	0.41	0.68	−0.14	0.22
	Female	247	4.85	0.73				
Self-compassion	Male	86	4.18	0.86	2.41	0.02	0.05	0.47
	Female	247	3.92	0.85				
Self-directedness	Male	86	4.64	0.80	1.69	0.09	−0.03	0.36
	Female	247	4.48	0.78				
Self-control	Male	86	4.48	0.80	2.95	<0.01	0.09	0.47
	Female	247	4.20	0.74				
CYPSQ total	Male	86	4.59	0.70	2.35	0.02	0.03	0.37
	Female	247	4.39	0.68				

*N* = 333. CYPSQ, Chinese version of the Young Positive Schema Questionnaire.

**TABLE 3:** Concurrent validity of the 11-factor Chinese version of the Young Positive Schema Questionnaire (CYPSQ).

	1	2	3	4	5	6	7	8	9	10	11	12	13	14	15	16
1. Age																
2. Fulfillment	0.23**															
3. Belonging	0.35**	0.76**														
4. Openness	0.38**	0.72**	0.81**													
5. Success	0.49**	0.64**	0.73**	0.69**												
6. Competence	0.55**	0.63**	0.72**	0.71**	0.80**											
7. Attachment	0.35**	0.73**	0.71**	0.66**	0.67**	0.66**										
8. Safety	0.45**	0.67**	0.73**	0.71**	0.74**	0.76**	0.76**									
9. Care	0.24**	0.74**	0.67**	0.65**	0.63**	0.66**	0.57**	0.60**								
10. Self-compassion	0.42**	0.51**	0.60**	0.62**	0.64**	0.63**	0.61**	0.73**	0.52**							
11. Self-directedness	0.39**	0.70**	0.70**	0.70**	0.74**	0.75**	0.67**	0.70**	0.76**	0.62**						
12. Self-control	0.40**	0.59**	0.67**	0.65**	0.76**	0.76**	0.61**	0.69**	0.62**	0.54**	0.73**					
13. CYPSQ total	0.46**	0.83**	0.87**	0.86**	0.87**	0.87**	0.83**	0.88**	0.80**	0.77**	0.87**	0.82**				
14. Resilience	0.28**	0.66**	0.68**	0.68**	0.70**	0.72**	0.61**	0.65**	0.68**	0.54**	0.70**	0.71**	0.79**			
15. Life satisfaction	0.16**	0.51**	0.52**	0.51**	0.50**	0.50**	0.43**	0.50**	0.45**	0.37**	0.46**	0.48**	0.56**	0.58**		
16. Anxiety	–0.01	−0.38**	−0.29**	−0.32**	−0.26**	−0.32**	−0.32**	−0.33**	−0.37**	−0.26**	−0.35**	−0.28**	−0.37**	−0.36**	−0.27**	
17. Depression	–0.08	−0.37**	−0.34**	−0.34**	−0.31**	−0.37**	−0.33**	−0.34**	−0.38**	−0.25**	−0.37**	−0.32**	−0.40**	−0.38**	−0.35**	0.84**

*N* = 329–336. CYPSQ, Chinese version of the Young Positive Schema Questionnaire.

**p < 0.01 (2-tailed).

### Construct validity

#### Assessing the fit of the model

The Kaiser–Meyer–Olkin measure of sampling adequacy was 0.97, and Bartlett’s test for sphericity was significant (chi-square = 13,502.89, *p* < 0.001), indicating appropriateness for factor analysis. The construct validities of the 14- and 1-factor CYPSQ were examined using CFA.

The fit indices of the 14-factor CYPSQ were χ^2^/df = 2.20, SRMR = 0.05, RMSEA = 0.06, GFI = 0.74, CFI = 0.87, and TLI = 0.86. Modifications were made to improve the construct. First, items with factor loadings lower than 0.60 (Items 4, 36, 2, 1, 19, 13, and 32) were removed. Then, the CFA was run again, the factor of realistic expectation (Items 10 and 53) was highly correlated with the factors of emotional fulfillment, self-care, and self-direction (correlation coefficient: 1.03, 1.05, and 1.06, respectively), and was thus removed. According to theories and data, self-reliance and boundaries (each factor with only two items and highly correlated with a correlation coefficient of 0.92) were combined and renamed as self-reliance and boundaries. The CFA was re-run and the factor of empathic consideration was combined with self-care (*r* = 0.95) and renamed as care. Items 8 and 42 were removed owing to covariance with many other items according to modification indices. Item 25 was removed owing to loadings lower than 0.6 (0.59). Finally, an 11-factor model with 44 items was obtained (Table 2). The fit indices were χ^2^/df = 2.13, SRMR = 0.04, RMSEA = 0.06, GFI = 0.80, TLI = 0.90, and CFI = 0.91, which were acceptable (Hu and Bentler, 1999).

The fit indices of the 1-factor CYPSQ with all 56 items were χ^2^/df = 2.81, SRMR = 0.05, RMSEA = 0.07, GFI = 0.64, CFI = 0.79, and TLI = 0.78. To improve the construct of the 1-factor CYPSQ, 11 items with factor loadings lower than 0.60 (Items 4, 36, 2, 1, 19, 12, 17, 13, 20, 22, and 32) were removed. The fit indices of this 1-factor construct with 45 items were χ^2^/df = 3.05, SRMR = 0.05, RMSEA = 0.08, GFI = 0.68, CFI = 0.82, and TLI = 0.81, which were less acceptable than the 11-factor construct (Hu and Bentler, 1999).

#### Convergent validity

Table 4 summarizes the convergent validity indices. Standardized factor loadings ranged from 0.61 to 0.87. AVE values ranged from 0.48 to 0.65, and most of them were above 0.50, which were considered adequate (Fornell and Larcker, 1981). Composite reliability values ranged from 0.73 to 0.90, which were all above the criterion of 0.70.

**TABLE 4:** Convergent validity and internal reliability of the 11-factor Chinese version of the Young Positive Schema Questionnaire (CYPSQ).

Factors	Item	Loadings	Cronbach’s α	AVE	CR	√A*VE*
1. Fulfillment	30	0.69	0.79	0.55	0.78	0.74
	50	0.74				
	55	0.79				
2. Belonging	3	0.68	0.87	0.58	0.87	0.76
	38	0.78				
	44	0.79				
	47	0.84				
	49	0.71				
3. Openness	9	0.75	0.87	0.65	0.88	0.80
	23	0.82				
	40	0.78				
	46	0.86				
4. Success	5	0.74	0.89	0.63	0.90	0.80
	15	0.80				
	35	0.74				
	43	0.82				
	48	0.87				
5. Self-reliance and boundaries	6	0.71	0.88	0.65	0.88	0.81
	41	0.87				
	45	0.87				
	24	0.77				
6. Attachment	28	0.82	0.79	0.57	0.80	0.76
	33	0.70				
	37	0.75				
7. Safety	7	0.64	0.85	0.54	0.86	0.74
	16	0.76				
	27	0.76				
	29	0.73				
	34	0.79				
8. Care	52	0.80	0.83	0.56	0.83	0.75
	56	0.81				
	20	0.68				
	51	0.69				
9. Self- compassion	12	0.61	0.72	0.48	0.73	0.69
	17	0.70				
	39	0.76				
10. Self- directedness	14	0.66	0.79	0.49	0.79	0.70
	21	0.68				
	26	0.75				
	54	0.70				
11. Self-control	11	0.76	0.79	0.48	0.79	0.70
	18	0.70				
	22	0.67				
	31	0.65				

*N* = 329–336. CYPSQ, Chinese version of the Young Positive Schema Questionnaire; AVE, average variance extracted; CR, composite reliability.

#### Discriminant validity

As shown in Table 5, discriminant validity was satisfied in most cases as the correlation coefficient for each factor was smaller than the root square of the AVE.

**TABLE 5:** Discriminant validity of the 11-factor Chinese version of the Young Positive Schema Questionnaire (CYPSQ).

Factors	1	2	3	4	5	6	7	8	9	10	11
1. Fulfillment	**0.74**										
2. Belonging	0.75	**0.76**									
3. Openness	0.72	0.81	**0.80**								
4. Success	0.64	0.74	0.69	**0.80**							
5. SRB	0.63	0.73	0.72	0.80	**0.81**						
6. Attachment	0.73	0.71	0.67	0.67	0.66	**0.76**					
7. Safety	0.67	0.73	0.72	0.75	0.76	0.76	**0.74**				
8. Care	0.74	0.70	0.66	0.63	0.66	0.58	0.60	**0.75**			
9. Self-compassion	0.52	0.61	0.63	0.65	0.63	0.61	0.73	0.53	**0.69**		
10. Self-directedness	0.70	0.70	0.71	0.74	0.75	0.67	0.70	0.76	0.63	**0.70**	
11. Self-control	0.60	0.67	0.65	0.76	0.76	0.62	0.70	0.62	0.55	0.73	**0.70**

*N* = 329–336. CYPSQ, Chinese version of the Young Positive Schema Questionnaire; SRB, self-reliance and boundaries. Root square of average variance extracted (AVE) in bold on diagonals.

### Concurrent validity

Concurrent validity was tested by examining the correlations between factors of life satisfaction, resilience, anxiety, depression, and factors of the CYPSQ. All 11 CYPSQ factors were positively correlated with life satisfaction and resilience, and negatively correlated with anxiety and depression (Table 3).

### Internal reliability

Internal reliability was verified as the Cronbach’s α and composite reliability values of the 11 factors of CYPSQ ranged from 0.72 to 0.89 and 0.73 to 0.90, respectively (Table 4), and these were all above the criterion of 0.70 (Fornell and Larcker, 1981; Nunnally and Bernstein, 1994).

## Discussion

The concept and theories of early adaptive or positive schemas are important complements to the EMS theory. However, as the concept and measurement of positive schema were proposed and published only recently (Lockwood and Perris, 2012; Louis et al., 2018), the psychometric information of the CYPSQ in Mainland China is still lacking. Therefore, this study examined the psychometric properties of the CYPSQ in a community sample from Mainland China.

### Main findings

#### Demographics

The findings of this study show that older people reported higher levels of all the 11 positive schemas. Few published studies have reported on the relationship between age and positive schemas. Thus, no comparison could be made. However, negative schemas were reported as dynamic constructs and may fade or intensify owing to specific contexts throughout the duration of one’s life (Legra et al., 2017). It was possible that positive schemas of the general population were developed through the course of individuals’ lives. Replications are required to confirm this finding.

Women reported lower levels of success, competence, safety, self-compassion, and self-control than men. No significant gender differences were found on other CYPSQ factors. According to the authors’ knowledge, no published studies have reported gender differences in positive schemas. Thus, no further comparisons could be made. However, a study among people with eating disorders and alcohol and substance use disorders found that women reported higher levels of maladaptive schemas such as abandonment, defectiveness, social undesirability, failure, dependence, subjugation, and self-sacrifice than men (Pauwels et al., 2013). Another study among people with alcohol dependence reported higher levels of self-sacrifice in women (Janson et al., 2019). It seems that women are more likely to be other-directed rather than self-directed, and are less likely to pursue success and competence. Studies across various cultures will help understand the sociocultural influence on positive schemas.

#### Construct validity

The findings of this study verified good psychometric properties with regard to most aspects of validity and reliability of the CYPSQ among a sample of 336 Chinese community residents. The original 14-factor YPSQ was verified in community samples from Singapore, the US, and Malaysia. However, this study identified an 11-factor YPSQ with 44 items instead of the original 14-factor YPSQ with 56 items. Nine positive schemas were conceptually fully retained: fulfillment (Items 4 and 25 were removed), belonging, openness, success, attachment (Item 2 was removed), safety, self-compassion, self-directedness, and self-control. The three items of self-reliance and one from boundaries (Item 24) were combined and renamed as self-reliance and boundaries. Two items each from self-care (Items 52 and 56) and empathic consideration (Items 20 and 51) were combined and renamed as care. The realistic expectation was removed. Similarly, a 10-factor YPSQ with 41 items was identified in a German community sample (Paetsch et al., 2020). It seems that the construct of YPSQ might vary a bit across cultures.

In the present study, the model fit indices of the 11-factor YPSQ were acceptable, the convergent validity was confirmed in all 11 factors, and the divergent validity was evident in 7 factors. Some factors showed a slightly lower divergent validity as they described a group of similar schemas. For example, fulfillment, belonging, and openness were positive schemas referring to social connection and acceptance; and self-directedness and self-control were about reasonable limits (Louis et al., 2018). The findings regarding convergent and divergent validities were consistent with those from other studies (Louis et al., 2018; Paetsch et al., 2020).

#### Concurrent validity

The findings of this study show that people with higher levels of positive schemas are more resilient and satisfied with life, and report lower levels of anxiety and depression. Positive schemas explained extra variance in depression, anxiety, stress, global severity of psychopathology, life satisfaction, resilience, and self-efficacy after controlling for EMSs (Louis et al., 2018; Paetsch et al., 2020). The positive schemas showed potential for reducing psychopathological outcomes and increasing one’s wellbeing. Future studies (especially longitudinal ones) can investigate such a possibility in clinical samples.

#### Internal reliability

The internal reliability was verified as all the Cronbach’s α and composite reliability values were above 0.70, indicating sufficient internal reliability (Fornell and Larcker, 1981; Nunnally and Bernstein, 1994). Similarly, the Cronbach’s α in the original 14- and 10-factor German YPSQ were all above 0.70, indicating good internal reliability across several population groups (Louis et al., 2018; Paetsch et al., 2020).

### Implications and limitations

This study verified the validity and reliability of an 11-factor CYPSQ in a Chinese Mainland community sample. It is important to note that the evaluation of positive schema can balance the overt attention to psychopathology in clinical psychology. The findings of this study may contribute toward investigating the role of positive schemas in improving mental health and psychological wellbeing in Mainland China. Future studies are needed to examine the psychometric properties of the CYPSQ in clinical populations. Some limitations of this study require attention. As this study was cross-sectional, the test-retest reliability and predictive validity could not be verified. This study was conducted in a convenient community sample, and the results may not represent the general population in Mainland China.

## Conclusion

This study verified the validity and reliability of the 11-factor CYPSQ in a Chinese community sample. Older people reported higher levels of all positive schemas than younger people, and men showed higher levels of some positive schemas (i.e., success, competence, safety, self-compassion, and self-control) than women. The findings show that positive schema levels may vary across population groups with different demographics (e.g., age and gender). This study may promote quantitative research on the role of positive schemas in improving mental health in Mainland China. The potentials of positive schemas in improving mental health and wellbeing need further longitudinal investigations.

## Data availability statement

The raw data supporting the conclusions of this article will be made available by the authors, without undue reservation.

## Ethics statement

The studies involving human participants were reviewed and approved by the Institutional Review Board of Ningbo Kangning Hospital (NBKNYY-2021-LC-50). The patients/participants provided their written informed consent to participate in this study.

## Author contributions

DC and YZ contributed to study design, advised on methodology, reviewed and edited the thesis and manuscript, and finalized the manuscript. HM, YW, and DC reviewed and revised the Chinese version of YPSQ. XD, YW, and HZ analyzed the data. HM, XD, and HZ prepared the original draft of the manuscript. All authors have read and agreed to the published version of the manuscript.
